# Looking back: Identifying supportive care and unmet needs of parents of children receiving specialist paediatric palliative care from the bereavement perspective

**DOI:** 10.1186/s12904-022-00971-y

**Published:** 2022-05-25

**Authors:** Annika Bronsema, Tabea Theißen, Karin Oechsle, Julia Wikert, Gabriele Escherich, Stefan Rutkowski, Carsten Bokemeyer, Anneke Ullrich

**Affiliations:** 1 Clinic of Paediatric Haematology and Oncology, University Medical Center Hamburg-Eppendorf, Martinistr. 52, 20246 Hamburg, Germany; 2 Palliative Care Unit, Department of Oncology, Hematology and BMT, University Medical Center Hamburg-Eppendorf, Hamburg, Germany; 3 Department of Palliative Medicine, University Hospital LMU, Munich, Germany

**Keywords:** Children, Parents, Palliative care, Supportive care needs, Cancer, Support services, FIN-PED II

## Abstract

**Background:**

This study examined care needs and utilisation of psychosocial support services among parents of children who had received specialist paediatric palliative care, as well as the relationship between need fulfilment and grief. Possible differences between parents of children who died of cancer versus a non-cancer disease were explored.

**Methods:**

This exploratory study, conducted in two specialist paediatric palliative care facilities, included parents who had lost a child within a period of 0.5 to 8 years before this investigation. From the bereavement perspective, parents reported their needs during paediatric palliative care using the Family Inventory of Needs – Peadiatric II (FIN-PED II). Utilisation of psychosocial support services during paediatric palliative care and after the child’s death, as well as potential barriers to accessing services were assessed. Grief symptoms were measured using the Inventory of Complicated Grief - German Version (ICG-D).

**Results:**

Overall, 56 of 157 approached parents participated in the study. Mean time interval after the child’s death was 3.2 years. Of the 17 FIN-PED II needs, 13 needs were reported to be very/extremely important to more than 75% of the parents each. Highest ranked needs related to asking questions at any time (100%), sincere care for the child (100%), and information about changes in the child’s condition (98%). The highest ranked unmet needs related to hope (61%), interactions with siblings (41–42%), and trust in the health care system (39%). Comparisons showed no significant differences between parents whose child died of cancer (*n* = 18) versus a non-cancer disease (*n* = 38). During paediatric palliative care, 61% of the parents had accessed at least one psychosocial support service and 84% had done so after the child’s death. The most prominent barriers for accessing services were sufficient informal support (38%), no subjective need (23%), and lack of time (20%). Overall, 52% of the parents showed noticeable symptoms for complicated grief (ICG-D > 25). A higher level of grief symptoms significantly correlated with a lower fulfilment of the need to say goodbye to the child (*p* = .042) with a medium correlational effect.

**Conclusions:**

Our findings may help to guide health care professionals in their assessment of parental needs and provision of support to parents during paediatric palliative care.

## Background

A life-limiting, incurable disease of a child is associated with manifold parental challenges, problems and needs, both before and after the child’s death [1].

Findings support that one in two parents taking care of a child with a life-limiting disease might meet the criteria for one or more diagnoses of clinically elevated levels of stress, anxiety or depression during caregiving [2]. Research shows that these parents experience difficulties in leaving care to others, so that relief of possible strain is even harder to establish [3]. Nevertheless, a review including 44 studies (2008–2019) on the support needs of parents of children with a life-limiting disease, identified support for respite care, out of hours, psychological, home and educational support as unmet needs [4]. Further, sibling support has been reported as a relevant area of parental needs in paediatric palliative care [5, 6]. Yet, the abovementioned review identified support for siblings as another unmet need among parents [4].

The loss of a child has tragic implications upon parents. Bereaved parents who cared for a child with a life-limiting disease have an elevated risk of anxiety, depression, deteriorated quality of life as well as prolonged grief [7, 8]. Posttraumatic stress syndrome can be observed in 20% of mothers and 35% of fathers of deceased children [9]. Risk factors for long-term psychosocial morbidities include psychiatric comorbidities, previous experiences of loss, financial burdens, duration and intensity of (cancer) therapies, preparation for and the place of the child’s death [8, 10].

Specialist paediatric palliative care should cover not only the needs of the ill children, but also those of their parents, siblings, and other significant family members [11]. Most child deaths (around 60%) in Germany occur within the first year of life, two thirds of which occur within the first 4 weeks [12]. According to German law, parents of children in need of specialist paediatric palliative care can choose between assistance at home, in hospitals (either non-palliative or palliative care wards) or as inpatients in hospices. In 2007, the German government implemented specialist outpatient palliative care into the health care system, and services specialised in paediatric care are available in most regions [13, 14]. The provision of paediatric palliative care is characteristically required by a heterogeneous population of children with life-limiting diseases [15] with more than 70% of the children suffering from non-cancer palliative diseases [13, 15, 16]. Previous research has shown that parents of the latter typically report more unmet needs with regards to information, as well as service provision and responsiveness compared to those caring for a child with cancer [17, 18]. Another study indicated that almost all parental needs investigated were more pronounced in parents of children with non-cancer diseases [19].

Research is still limited on the support needs of parents of children with a life-limiting disease, especially in the context of specialist paediatric palliative care. Furthermore, few studies use valid and reliable instruments to assess parental needs [4]. Therefore, we conducted this exploratory study with bereaved parents of children who had died and had received specialist paediatric palliative care. The primary aim was to examine supportive care needs as well as the utilisation of psychosocial support services among these parents using a retrospective survey. We further investigated the relationship between parental grief and need fulfilment, given that a previous study indicated a correlation between grief symptoms and unmet needs among parents of children who died in the intensive care unit [20]. A secondary aim was to compare parents of children who had died from cancer versus non-cancer diseases with regards to these aspects, as studies indicate that needs could vary between these groups [17–19].

## Methods

### Study design and participants

This cross-sectional, observational, prospective study was conducted between March 2019 and July 2020 in the context of specialist paediatric palliative care in Hamburg, Germany. Participants were recruited from the Clinic of Paediatric Haematology and Oncology, University Medical Centre Hamburg-Eppendorf, as well as from the only specialist paediatric outpatient palliative care team in Hamburg. Inclusion criteria were: being a parent with custody of a child who died between January 2011 and December 2019 and had received specialist paediatric palliative care from one of the abovementioned institutions (minimum 6 months after the child’s death), age of 18 years and cognitive capacity for giving fully informed consent and completing the questionnaire. The exclusion criteria were: death due to treatment related mortality, immediate postnatal death, unknown cause of death.

The coordinator of the specialist outpatient paediatric palliative care team and a senior consultant (AB) identified eligible parents from the institution’s databases. One questionnaire per child, as well as a study information and the informed consent form were sent to eligible parents by post. Since we were aware of potentially changed living conditions after child loss (e.g. due to the heightened risk of parental separation and/or divorce, as indicated by evidence [21]), we asked parents to self-identify the responding person.

Those parents, who were notified about the study but did not respond, received a single written reminder after 8 weeks. After that, there was no further contacting, and non-respondents were considered as not wanting to participate (passive refusal). Written informed consent was obtained from all participating parents. The ethics committee of the Medical Association in Hamburg, Germany approved the study (October 02, 2018; Reference number: PV5967).

### Instruments

#### Supportive care needs

The perceived care needs of parents and the extent to which these needs were met were measured using the multidimensional *Family Inventory of Needs-Paediatrics II* (FIN-PED II) [17]. With explicit permission of the authors, the original FIN-PED II was translated into German, in a culturally adapted standard forward- and back-translation process with monolingual and bilingual tests [22]. In addition, the translations were compared to the validated German version of the *Family Inventory of Needs* (FIN), frequently used in adult palliative care [23]. The FIN-PED II contains 17 items, each of which is rated on three subscales: “Importance of Care Needs” (0 “not at all important” to 4 “extremely important”), “Need Fulfilment” (0 “no need”, 1 “not met at all” to 4 “completely met”) and “Need for Further Information” (0 “no further information required” to 4 “a great deal of information”). Reliability and validity have been supported for the original FIN-PED II [24]. Cronbach’s alpha for the German FIN-PED II in this study was .82 for “Importance of Care Needs”, .80 for “Need Fulfilment” and .95 for “Need for Further Information”.

Additionally, a focus group of multiprofessional experts working in specialist paediatric palliative care (two physicians, two nurses, one psychologist and one music therapist) discussed needs of parents as experienced during their clinical encounters. In a first phase, the focus group developed seven additional need items. Additionally, items from existing questionnaires on parent caregiver’s needs [20, 25, 26] were prioritized by this group of experts. In this second phase, the focus group identified nine additional need items. Thus 16 need items were added to the FIN-PED II questionnaire. Cronbach’s alpha for the extended version, including the added items, was .90 for “Importance of Care Needs”, .89 for “Need Fulfilment” and .98 for “Need for Further Information”.

#### Grief

Complicated grief was measured using the 19-item *Inventory of Complicated Grief - German Version* (ICG-D) [27]. The ICG-D is validated and based on the *Inventory of Complicated Grief* developed by Prigerson et al. [28]. It measures 19 different grief-related symptoms. The frequency of the grief-related state is rated on a scale from 0 (never) to 4 (always). The total score ranges from 0 to 76, with higher values representing higher levels of complicated grief symptoms. The authors defined a total score of 25 as a cut-off, values above this cut-off indicate noticeable grief symptoms [28]. Cronbach’s alpha for the ICG-D in this study was .90.

#### Utilisation of support services

Parents reported their use of psychosocial support services during paediatric palliative care and after the child’s death, as well as potential barriers to access existing support services. The support services included self-support groups, cancer-counselling services, psychological, legal and spiritual counselling, counselling of parenting and family issues, and bereavement care. The items were already used in previous research about family caregivers of advanced cancer patients [29].

#### Sociodemographic and medical variables

For demographic characteristics, information was collected on parents` age, gender, marital status and partnership, place of birth, religion and level of education. The parents` level of education was assessed using the International Standard Classification of Education (ISCED) [30]. These levels range from 0 to 8 and are categorized into low (levels 0–2), medium (levels 3–4) and high (levels 5–8) education. In addition, parents reported on their child’s age at death, gender, time since death, diagnosis, and number of siblings.

#### Data analysis

Descriptive statistics were calculated including the frequencies, percentages, means and standard deviations (SD).

The needs of the three FIN-PED II subscales were analysed on a single item level in congruence with previous studies in this field using this instrument [24, 31]. A mean score from each item that are rated between 1 and 4 was calculated. Needs rated as 3 (“very much”) or 4 (“extremely”) on the “Importance of Care Needs” scale were considered important needs. On the “Need Fulfilment” scale, needs rated as 1 (“not met at all”) or 2 (“partly met”) were considered unmet.

Complicated grief was analysed using the mean total score of the ICG-D. Missing data was handled using the expectation-maximization algorithm (EM-algorithm). One case was excluded due to more than 10% missing values. In two cases there were less than 10% missing, those were substituted using the EM-algorithm.

All computations were performed for the total sample and for parents of children who died from cancer versus a non-cancer disease. Fisher’s exact tests (for categorical variables) and two-sample t-tests (for continuous variables) were used to compare groups. The Fisher’s test was conducted when the expected cell counts were below five for at least one cell.

Finally, Pearson’s correlation coefficient was used to test whether the total score of the ICG-D and the “Need Fulfilment” scale of the FIN-PED II correlate with each other. The effect size was interpreted in accordance to Cohen [32]: *d* = 0.2 is considered a small effect, *d* = 0.5 a moderate effect and *d* = 0.8 represents a large effect.

Due to the exploratory approach of the study, no adjustments were made for multiple comparisons [33, 34]. All statistical analyses were computed using IBM SPSS Statistics version 26.0 [35] and a significance level of α < 0.05.

## Results

### Recruitment procedure

**Fig. 1:**
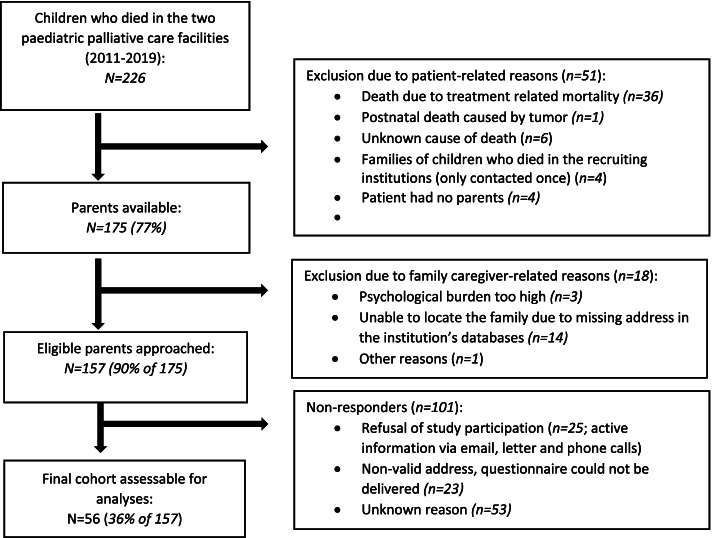
Study recruiting process and sample development

Within the investigated period, 226 children had died, 127 from the Clinic of Paediatric Haematology and Oncology and 99 from the specialist paediatric outpatient palliative care team. Of these, 51 parents were excluded due to patient-related reasons and 18 due to caregiver-related reasons. Thus 157 parents were approached and 56 returned a questionnaire (response rate: 36%). Details are shown in Fig. 1.

### Characteristics of bereaved parents

Table 1 summarizes and compares the demographic characteristics of the participating parents. Their mean age was 44.4 years (*SD* 7.5, range 29–57) and 57% (*n* = 32) were highly educated. The balance between mothers and fathers was unequally distributed with most participants (75%) being mothers. On average, children had died 3.2 years ago (*SD* 2.2, range 0.5–8) and were aged 7.3 years (*SD* 6.9, range 0–19) at the time of their death. Parents of children with a non-cancer disease were more likely to be mothers (*p =* .044) and more frequently married (*p =* .040). In addition, children with a non-cancer disease were younger at their death (*p =* .001).

**Table 1:** Parent- and children-related characteristics (*N = 56*) and comparison of the parents of children with cancer (*N = 18*) and those of children with a non-cancer disease (*N = 38*)

*Family caregiver characteristics*		Overall (***N*** = 56)	Parents of children with cancer (***N*** = 18)	Parents of children with a non-cancer disease (***N*** = 38)	***p***-value
Relation to the child, *n* (%)	Mother	42 (75)	10 (55.6)	32 (84.2)	.044* ^a^
	Father	14 (25)	8 (44.4)	6 (15.8)	
Age, *M* (*SD*); Range		44.4 (7.5);29–57	47.2 (6.1);37–55	43.1 (7.7);29–57	.051 ^b^
Age in Groups, *n* (%)	29–39	16 (28.6)	3 (16.7)	13 (34.2)	.183 ^a^
	40–49	24 (42.9)	7 (38.9)	17 (44.7)	
	50–59	16 (28.6)	8 (44.4)	8 (21.2)	
Marital status, *n* (%)	Single	3 (5.4)	0 (0)	3 (7.9)	.040* ^a^
	Married	42 (75)	11 (61.1)	31 (81.6)	
	Divorced or widowed	11 (19.6)	7 (38.9)	4 (10.5)	
Partnership,* n* (%)	Yes	45 (80.4)	12 (66.7)	33 (86.8)	.224 ^a^
	No	8 (14.3)	4 (22.2)	4 (10.5)	
	Missing	3 (5.4)	2 (11.1)	1 (2.6)	
Religion, *n* (%)	Yes	37 (66.1)	11 (61.1)	26 (68.4)	.763 ^a^
	No	19 (33.9)	7 (38.9)	12 (31.6)	
Place of Birth, *n* (%)	Germany	47 (83.9)	14 (77.8)	33 (86.8)	.205 ^a^
	Other	7 (12.5)	4 (22.2)	3 (7.9)	
	Missing	2 (3.6)	0 (0)	2 (5.3)	
Level of Education,* n* (%)	ISCED Low	2 (3.6)	1 (5.6)	1 (2.6)	.188 ^b^
	ISCED Middle	22 (39.3)	9 (50)	13 (34.2)	
	ISCED High	32 (57.1)	8 (44.4)	24 (63.2)	
*Children-related characteristics*					
Child gender, *n* (%)	Female	31 (55.4)	8 (44.4)	23 (60.5)	.388 ^a^
	Male	25 (44.6)	10 (55.6)	15 (39.5)	
Child age at death (in years), *M* (*SD*); Range		7.3 (6.9);0–19	11.9 (5.4);2–19	5.0 (6.5);0–19	.001** ^b^
Child age at death categorized (in years),* n* (%)	≤3	27 (48.2)	2 (11.1)	25 (65.8)	.001** ^a^
	4–7	4 (7.1)	2 (11.1)	2 (5.3)	
	8–11	7 (12.5)	5 (27.8)	2 (5.3)	
	12–15	7 (12.5)	2 (11.1)	5 (13.2)	
	> 15	11 (19.6)	7 (38.9)	4 (10.5)	
Time since death, *M* (*SD*); Range		3.2 (2.2);0.5–8	3 (1.9);0.5–6	3.2 (2.3);0.5–8	.707 ^b^
Time since death in years, categorized, *n* (%)	< 1	6 (10.7)	2 (11.1)	4 (10.5)	1.00 ^a^
	1–4	33 (58.9)	11 (61.1)	22 (57.9)	
	≥5	17 (30.4)	5 (27.8)	12 (31.6)	
Disease, *n *(%)	Cancer	18 (32.1)	–	–	–
	Non-cancer	38 (67.9)	–	–	
Cancer diagnosis,* n* (%)	Leukaemia	–	3 (16.7)	–	–
	Brain tumor	–	9 (50.0)	–	
	Solid Tumor	–	6 (33.3)	–	
Non-cancer diagnosis, *n* (%)	Conditions, where premature death in inevitable, with intensive treatment (e.g., muscular dystrophy)	–	–	5 (13.2)	–
	Progressive disease without curative treatment options (e.g., MPS, NCL)	–	–	10 (26.3)	
	Irreversible non-progressive conditions, causing severe neurological disorder	–	–	23 (60.5)	
Siblings, *n* (%)	0	6 (10.7)	1 (5.6)	5 (13.2)	.089 ^b^
	1	26 (46.4)	12 (66.7)	14 (36.8)	
	2	17 (30.4)	4 (22.2)	13 (34.2)	
	3	6 (10.7)	1 (5.6)	5 (13.2)	
	Missing	1 (1.8)	0 (0)	1 (2.6)	

*Abbreviations*: *M* Mean, *SD* Standard deviation, *ISCED* International Standard Classification of Education (UNESCO, 2012), *MPS* Mucopolysaccharide diseases, *NCL* Neuronal Ceroid Lipofuscinosis

^a^ Fisher’s Exact Test, ^b^ t-test

* Significance at *p* < 0.05. ** Significance at *p* < 0.01

### Importance of care needs

Overall, 13 of the 17 FIN-PED II needs were rated as very/extremely important by more than 75% of the parents, as shown in Table 2. The range of means was 2.6 to 3.9, on a scale of 0 to 4, with standard deviations between 0.4 and 1.5. The highest ranked needs were *asking questions any time* (*M* = 3.9*,* 100% very/extremely important), *feeling that the health care professionals were sincere about the child’s care* (*M* = 3.8*,* 100%), *being informed about the child’s condition* (*M* = 3.8, 98%) and *knowing how to care for the child at home* (*M* = 3.7*,* 96%). The lowest ranked needs were *feeling hope* (*M* = 2.6, 59%), *knowing how and what information to give to the child’s siblings* (both *M* = 2.8, 72–74%), and *having trust in the health care system* (*M* = 2.8, 73%). Comparisons showed no significant differences between parents of a child with cancer and those of a child with a non-cancer disease.

**Table 2:** Importance of parent’s needs (*N* = 56) and comparison of parents of children with cancer (*N* = 18) and those of children with a non-cancer disease (*N* = 38)

FIN-PED II Importance of Care Needs	Overall (***N*** = 56)		Parents of children with cancer (***N*** = 18)		Parents of children with a non-cancer disease (***N*** = 38)		*p*-value
	Importance of Care Needs	Very/extremely important ^a^	Importance of Care Needs	Very/extremely important ^a^	Importance of Care Needs	Very/extremely important ^a^	
I needed:	*M (SD)*	*n/N* (%)	*M (SD)*	*n/N* (%)	*M (SD)*	*n/N* (%)	
**1** To feel there was hope	2.6 (1.4)	31/53 (58.5)	2.9 (1.4)	13/18 (72.2)	2.5 (1.3)	18/35 (51.4)	.239 ^b^
**2** To know *when* to expect side effects to occur	3.1 (1.0)	44/54 (81.5)	2.9 (1.2)	12/18 (66.7)	3.2 (0.9)	32/36 (88.9)	.067 ^b^
**3** To know *what* side effects the treatment can cause	3.3 (0.8)	47/54 (87.0)	3.1 (1.0)	15/18 (83.3)	3.4 (0.7)	32/36 (88.9)	.674 ^b^
**4** To have thorough information about how to care for my child at home	3.7 (0.6)	53/55 (96.4)	3.4 (0.9)	16/18 (88.9)	3.9 (0.3)	37/37 (100)	.103 ^b^
**5** To know that health care professionals offer me the opportunity to participate equally in my child’s care	3.6 (0.9)	52/55 (94.5)	3.5 (1.0)	16/18 (88.9)	3.7 (0.8)	36/37 (97.3)	.247 ^b^
**6** To have trust in the health care system	2.8 (1.1)	41/56 (73.2)	2.9 (0.7)	13/18 (72.2)	2.8 (1.2)	28/38 (73.7)	1.00 ^b^
**7** To be informed of changes to my child’s condition	3.8 (0.5)	54/55 (98.2)	3.7 (0.6)	17/18 (94.4)	3.8 (0.4)	37/37 (100)	.327 ^b^
**8** To know what treatment my child was receiving	3.8 (0.5)	53/55 (96.4)	3.7 (0.6)	17/18 (94.4)	3.8 (0.4)	36/37 (97.3)	1.00 ^b^
**9** To feel that the health care professionals were sincere in caring about my child	3.9 (0.3)	56/56 (100)	3.8 (0.4)	18/18 (100)	3.9 (0.3)	38/38 (100)	n.a.^c^
**10** To have explanations given in terms that were understandable to me	3.4 (0.8)	47/55 (85.5)	3.4 (0.8)	15/18 (83.3)	3.4 (0.9)	32/37 (86.5)	1.00 ^b^
**11** To be told when and why changes were being made in my child’s treatment plans	3.5 (0.9)	50/53 (94.3)	3.3 (1.0)	15/17 (94.4)	3.6 (0.8)	35/36 (97.2)	.238 ^b^
**12** To know I could ask questions any time	3.8 (0.4)	55/55 (100)	3.8 (0.4)	18/18 (100)	3.8 (0.4)	37/37 (100)	n.a. ^c^
**13** To know to whom I should direct my questions	3.6 (0.6)	51/55 (92.7)	3.6 (0.6)	17/18 (94.4)	3.6 (0.6)	34/37 (91.9)	1.00 ^b^
**14** To know the probable outcome of my child’s illness	3.7 (0.8)	52/56 (92.9)	3.6 (0.7)	16/18 (88.9)	3.7 (0.8)	36/38 (94.7)	.587 ^b^
**15** To know *how* to give information to my other children (appropriate to his/her age)	2.8 (1.5)	34/47 (72.3)	3.0 (1.5)	15/18 (83.3)	2.7 (1.6)	19/29 (65.5)	.315 ^b^
**16** To know *what* information to give to my other children (appropriate to his/her age)	2.8 (1.5)	34/46 (73.9)	3.0 (1.5)	15/18 (83.3)	2.7 (1.6)	19/28 (67.9)	.315 ^b^
**17** To know how to handle the feelings of my other children	3.1 (1.4)	37/46 (80.4)	3.1 (1.5)	15/18 (83.3)	3.0 (1.4)	22/28 (78.6)	1.00 ^b^
**Importance of Care Needs – additional items**							
**18** To have confidence in the staff caring for my child	3.9 (0.4)	55/55 (100)	3.9 (0.2)	18/18 (100)	3.8 (0.4)	37/37 (100)	n.a. ^c^
**19** To know that my child’s pain is well adjusted	3.9 (0.3)	55/55 (100)	3.9 (0.3)	18/18 (100)	4.0 (0.2)	37/37 (100)	n.a. ^c^
**20** To be able to speak with my child’s treating physician at any time	3.5 (0.7)	49/55 (89.1)	3.7 (0.6)	17/18 (94.4)	3.5 (0.7)	32/37 (86.5)	.651 ^b^
**21** To have enough time to make decisions	3.3 (1.0)	48/54 (88.9)	3.1 (1.2)	16/18 (88.9)	3.4 (0.9)	32/36 (88.9)	1.00 ^b^
**22** To have my child be treated with dignity	3.9 (0.3)	56/56 (100)	3.9 (0.2)	18/18 (100)	3.9 (0.3)	38/38 (100)	n.a. ^c^
**23** To have the opportunity to say goodbye to my child	4.0 (0.2)	56/56 (100)	3.9 (0.2)	18/18 (100)	4.0 (0.2)	38/38 (100)	n.a. ^c^
**24** To be with my child when he/she dies	3.9 (0.3)	53/54 (98.1)	3.9 (0.2)	18/18 (100)	3.9 (0.4)	35/36 (97.2)	1.00 ^b^
**25** To reach the team 24 hours a day, 7 days a week	3.8 (0.5)	55/56 (98.2)	3.8 (0.4)	18/18 (100)	3.7 (0.5)	37/38 (97.4)	1.00 ^b^
**26** To be prepared for the medical aspects of dying by the treating team	3.5 (0.7)	49/55 (89.1)	3.3 (0.9)	15/18 (83.3)	3.5 (0.7)	34/37 (91.9)	.381 ^b^
**27** To know that my other children were being well cared for as well	3.2 (1.3)	38/46 (82.6)	3.1 (1.3)	14/18 (77.8)	3.2 (1.3)	24/28 (85.7)	.693 ^b^
**28** To have a contact person for every situation at hand	3.4 (0.8)	49/54 (90.7)	3.6 (0.6)	17/18 (94.4)	3.4 (0.9)	32/36 (88.9)	.655 ^b^
**29** To have room for conversations with the team about issues other than my child’s disease	2.3 (1.3)	23/55 (41.8)	2.4 (1.3)	11/18 (61.1)	2.2 (1.4)	12/37 (32.4)	.079 ^b^
**30** To be respected for the way I deal with my child’s disease	3.5 (0.9)	49/55 (89.1)	3.4 (1.0)	16/18 (88.9)	3.5 (0.8)	33/37 (89.2)	1.00 ^b^
**31** To get support in dealing with different treatment requests within our family	2.5 (1.3)	31/54 (57.4)	2.5 (1.4)	12/18 (66.7)	2.5 (1.3)	19/36 (52.8)	.392 ^b^
**32** To have enough time for myself, e.g. for relaxation	1.9 (1.4)	17/55 (30.9)	2.4 (1.4)	9/18 (50.0)	1.6 (1.3)	8/37 (21.6)	.060 ^b^
**33** To get advice on issues of social-law, such as cost assumption	2.8 (1.2)	38/55 (69.1)	2.8 (1.2)	13/18 (72.2)	2.7 (1.2)	25/37 (67.6)	1.00 ^b^

Abbreviations: *M* Mean, *SD* Standard deviation

^a^ versus not/a little/somewhat important

^b^ Fisher’s exact test, ^c^ not applicable

*p*-values are referring to proportions: * Significance at *p* < 0.05, ** Significance at *p* < 0.01

Importance of care needs rank: a little important (1) to extremely important (4)

Of the 16 additional needs, 12 were rated as very/extremely important by more than 75% of the parents. The range of means was 1.9 to 4.0 with standard deviations between 0.3 and 1.4. The highest ranked needs were *having the opportunity to say goodbye* (*M* = 4.0*,* 100% very/extremely important), *having confidence in the staff, having the child treated with dignity, knowing that the child’s pain is well adjusted,* and *being with the child in the moment of death* (each *M* = 3.9*,* 100%). The lowest ranked needs were *having enough time for yourself* (*M* = 1.9*,* 31%) and *having room for conversations about issues other than the child’s disease* (*M* = 2.3*,* 42%).

Comparisons between parents of children who died of cancer versus a non-cancer disease showed no significant differences in the need importance.

### Need fulfilment

In the total cohort of parents, two needs were unmet in more than 50%: *feeling hope* (*M* = 1.8, 61% unmet) and *knowing how to handle the siblings’ feelings* (*M* = 2.0, 55%). The range of means was 1.8 to 3.5, on a scale of 1 to 4, with standard deviations between 0.7 and 1.3.

Of the 16 additional needs, three were unmet in more than one third of the parents: *having enough time for yourself* (*M* = 1.7, 44%), *getting advice on issues of social-law* (*M* = 2.5, 37%), and *being prepared for the medical aspects of dying* (*M* = 2.9, 35%). The range of means was 1.7 to 3.8 with standard deviations between 0.7 and 1.4. Details are shown in Table 3.

**Table 3:** Fulfilment of parent’s needs (*N* = 56) and comparison of parents of children with cancer (*N* = 18) and those of children with a non-cancer disease (*N* = 38)

FIN-PED II Need Fulfilment	Overall (***N*** = 56)		Parents of children with cancer (***N*** = 18)		Parents of children with a non-cancer disease (***N*** = 38)		*p*-value
	Need Fulfilment	not met at all/ partly met ^a^	Need Fulfilment	not met at all/ partly met ^a^	Need Fulfilment	not met at all/ partly met ^a^	
I needed:	*M (SD)*	*n/N* (%)	*M (SD)*	*n/N* (%)	*M (SD)*	*n/N* (%)	
**1** To feel there was hope	1.8 (1.2)	27/44 (61.4)	1.6 (1.1)	9/14 (64.3)	1.9 (1.2)	18/30 (60.0)	1.00 ^b^
**2** To know *when* to expect side effects to occur	2.7 (0.9)	16/52 (30.8)	2.8 (1.0)	4/17 (23.5)	2.7 (0.8)	12/35 (34.3)	.532 ^b^
**3** To know *what* side effects the treatment can cause	2.8 (0.8)	15/53 (28.3)	2.7 (1.0)	5/17 (29.4)	2.9 (0.6)	10/36 (27.8)	1.00 ^b^
**4** To have thorough information about how to care for my child at home	3.2 (0.8)	11/55 (20.0)	3.3 (0.7)	2/18 (11.1)	3.1 (0.8)	9/37 (24.3)	.307 ^b^
**5** To know that health care professionals offer me the opportunity to participate equally in my child’s care	3.3 (0.9)	6/53 (11.3)	2.9 (1.1)	4/17 (23.5)	3.4 (0.8)	2/36 (5.6)	.076 ^b^
**6** To have trust in the health care system	2.4 (1.1)	20/51 (39.2)	2.4 (1.0)	6/17 (35.3)	2.4 (1.1)	14/34 (41.2)	.767 ^b^
**7** To be informed of changes to my child’s condition	3.0 (0.8)	13/55 (23.6)	2.9 (0.7)	3/18 (16.7)	3.1 (0.8)	10/37 (27.0)	.510 ^b^
**8** To know what treatment my child was receiving	3.3 (0.7)	6/55 (10.9)	3.2 (0.7)	3/18 (16.7)	3.3 (0.7)	3/37 (8.1)	.381 ^b^
**9** To feel that the health care professionals were sincere in caring about my child	3.5 (0.7)	6/56 (10.7)	3.2 (0.8)	4/18 (22.2)	3.6 (0.6)	2/38 (5.3)	.077 ^b^
**10** To have explanations given in terms that were understandable to me	3.3 (0.8)	6/54 (11.1)	3.1 (0.6)	3/18 (16.7)	3.4 (0.9)	3/36 (8.3)	.388 ^b^
**11** To be told when and why changes were being made in my child’s treatment plans	3.1 (0.9)	6/51 (11.8)	2.8 (1.0)	3/16 (18.8)	3.2 (0.8)	3/35 (8.6)	.363 ^b^
**12** To know I could ask questions any time	3.4 (0.8)	10/55 (18.2)	3.1 (0.8)	5/18 (27.8)	3.6 (0.7)	5/37 (13.5)	.268 ^b^
**13** To know to whom I should direct my questions	3.2 (0.8)	12/55 (21.8)	3.0 (0.8)	5/18 (27.8)	3.3 (0.8)	7/37 (18.9)	.499 ^b^
**14** To know the probable outcome of my child’s illness	2.7 (1.0)	20/54 (37.0)	2.7 (0.8)	7/18 (38.9)	2.7 (1.0)	13/36 (36.1)	1.00 ^b^
**15** To know *how* to give information to my other children (appropriate to his/her age)	2.1 (1.3)	16/39 (41.0)	1.8 (1.3)	9/15 (60.0)	2.3 (1.2)	7/24 (29.2)	.094 ^b^
**16** To know *what* information to give to my other children (appropriate to his/her age)	2.0 (1.3)	16/38 (42.1)	1.8 (1.3)	8/15 (53.3)	2.2 (1.3)	8/23 (34.8)	.324 ^b^
**17** To know how to handle the feelings of my other children	2.0 (1.2)	22/40 (55.0)	1.7 (1.2)	11/15 (73.3)	2.2 (1.2)	11/25 (44.0)	.104 ^b^
**Need Fulfilment – additional items**							
**18 **To have confidence in the staff caring for my child	3.2 (0.7)	10/54 (18.5)	3.1 (0.7)	3/18 (16.7)	3.3 (0.8)	7/36 (19.4)	1.00 ^b^
**19** To know that my child’s pain is well adjusted	3.0 (0.8)	14/54 (25.9)	3.1 (0.7)	3/18 (16.7)	2.9 (0.8)	11/36 (30.6)	.339 ^b^
**20** To be able to speak with my child’s treating physician at any time	3.0 (0.8)	15/55 (27.3)	2.8 (0.6)	6/18 (33.3)	3.0 (0.9)	9/37 (24.3)	.688 ^b^
**21** To have enough time to make decisions	2.8 (1.0)	15/54 (27.8)	2.4 (1.1)	6/18 (33.3)	2.9 (0.9)	9/36 (25.0)	.251 ^b^
**22** To have my child be treated with dignity	3.5 (0.7)	6/55 (10.9)	3.4 (0.7)	2/18 (11.1)	3.6 (0.7)	4/37 (10.8)	1.00 ^b^
**23** To have the opportunity to say goodbye to my child	3.7 (0.7)	4/54 (7.4)	3.5 (0.6)	1/17 (5.9)	3.8 (0.7)	3/37 (8.1)	1.00 ^b^
**24** To be with my child when he/she dies	3.8 (0.7)	4/54 (7.4)	3.9 (0.3)	0/18 (0)	3.7 (0.8)	4/36 (11.1)	.289 ^b^
**25** To reach the team 24 hours a day, 7 days a week	3.6 (0.7)	3/55 (5.5)	3.5 (0.8)	1/18 (5.6)	3.7 (0.6)	2/37 (5.4)	1.00 ^b^
**26** To be prepared for the medical aspects of dying by the treating team	2.9 (1.0)	19/54 (35.2)	2.9 (0.9)	5/18 (27.8)	2.9 (1.0)	14/36 (38.9)	.550 ^b^
**27** To know that my other children were being well cared for as well	2.4 (1.4)	14/46 (30.4)	2.3 (1.4)	4/18 (22.2)	2.5 (1.5)	10/28 (35.7)	.643 ^b^
**28** To have a contact person for every situation at hand	2.9 (1.0)	12/54 (22.2)	2.9 (0.6)	4/18 (22.2)	2.9 (1.2)	8/36 (22.2)	.677 ^b^
**29** To have room for conversations with the team about issues other than my child’s disease	2.3 (1.3)	13/54 (24.1)	2.4 (1.3)	4/18 (22.2)	2.2 (1.4)	9/36 (25.0)	.927 ^b^
**30** To be respected for the way I deal with my child’s disease	3.2 (0.9)	8/55 (14.5)	3.0 (1.0)	3/18 (16.7)	3.4 (0.8)	5/37 (13.5)	.295 ^b^
**31** To get support in dealing with different treatment requests within our family	2.3 (1.3)	12/53 (22.6)	2.2 (1.3)	3/18 (16.7)	2.3 (1.3)	9/35 (25.7)	.789 ^b^
**32** To have enough time for myself, e.g. for relaxation	1.7 (1.3)	24/54 (44.4)	2.0 (1.3)	7/18 (38.9)	1.5 (1.3)	17/36 (47.2)	.537 ^b^
**33** To get advice on issues of social-law, such as cost assumption	2.5 (1.2)	20/54 (37.0)	2.7 (1.3)	4/18 (22.2)	2.5 (1.2)	16/36 (44.4)	.248 ^b^

Abbreviations: *M* Mean, *SD* Standard deviation

^a^ versus well/completely met, ^b^ Fisher’s exact test

*p*-values are referring to proportions: * Significance at *p* < 0.05, ** Significance at *p* < 0.01

Need Fulfilment rank: not met at all (1) to completely met (4)

Comparing parents of children who died of cancer versus a non-cancer disease, there was no significant difference.

### Need for further information

In the total cohort, four needs required very much/a great deal of further information for more than one third of the parents (Table 4). The range of means was 1.0 to 1.8, on a scale of 0 to 4, with standard deviations between 1.2 and 1.6. Highest ranked needs were needs related to *interactions with the child’s siblings* (*M* = 1.5–1.7, 37–41% very much/great deal), *knowing the probable outcome of the child’s illness* (*M* = 1.8, 40%), and *knowing when to expect side effects to occur* (*M* = 1.6, 36%).

**Table 4:** Parent’s need for further information (*N* = 56) and comparison of parents of children with cancer (*N* = 18) and those of children with a non-cancer disease (*N* = 38)

FIN-PED II Need for Further Information	Overall (***N*** = 56)		Parents of children with cancer (***N*** = 18)		Parents of children with a non-cancer disease (***N*** = 38)		*p*-value
	Need for Further Information	Very much/a great deal ^a^	Need for Further Information	Very much/a great deal ^a^	Need for Further Information	Very much/a great deal ^a^	
I needed:	*M (SD)*	*n/N* (%)	*M (SD)*	*n/N* (%)	*M (SD)*	*n/N* (%)	
**1** To feel there was hope	1.2 (1.2)	7/52 (13.5)	1.2 (1.3)	3/18 (16.7)	1.2 (1.2)	4/34 (11.8)	.682 ^b^
**2** To know *when* to expect side effects to occur	1.6 (1.4)	19/53 (35.8)	1.7 (1.2)	6/18 (33.3)	1.6 (1.6)	13/35 (37.1)	1.00 ^b^
**3** To know *what* side effects the treatment can cause	1.6 (1.5)	17/53 (32.1)	1.6 (1.5)	4/18 (22.2)	1.6 (1.6)	13/35 (37.1)	.358 ^b^
**4** To have thorough information about how to care for my child at home	1.3 (1.5)	14/50 (28.0)	1.2 (1.5)	4/17 (23.5)	1.4 (1.5)	10/33 (30.3)	.746 ^b^
**5** To know that health care professionals offer me the opportunity to participate equally in my child’s care	1.1 (1.3)	10/52 (19.2)	1.2 (1.3)	3/18 (16.7)	1.1 (1.2)	7/34 (20.6)	1.00 ^b^
**6** To have trust in the health care system	1.5 (1.4)	17/53 (32.1)	1.5 (1.5)	6/18 (33.3)	1.5 (1.4)	11/35 (31.4)	1.00 ^b^
**7** To be informed of changes to my child’s condition	1.6 (1.4)	14/50 (28.0)	1.8 (1.6)	7/17 (41.2)	1.5 (1.4)	7/33 (21.2)	.187 ^b^
**8** To know what treatment my child was receiving	1.3 (1.4)	11/52 (21.2)	1.4 (1.6)	4/17 (23.5)	1.3 (1.3)	7/35 (20.0)	1.00 ^b^
**9** To feel that the health care professionals were sincere in caring about my child	1.0 (1.2)	8/52 (15.4)	1.2 (1.3)	4/17 (23.5)	0.9 (1.2)	4/35 (11.4)	.413 ^b^
**10** To have explanations given in terms that were understandable to me	1.1 (1.2)	7/52 (13.5)	1.3 (1.3)	3/17 (17.6)	0.9 (1.2)	4/35 (11.4)	.670 ^b^
**11** To be told when and why changes were being made in my child’s treatment plans	1.2 (1.3)	7/50 (14.0)	1.5 (1.3)	3/17 (17.6)	1.0 (1.2)	4/33 (12.1)	.677 ^b^
**12** To know I could ask questions any time	1.2 (1.5)	11/52 (21.2)	1.6 (1.4)	4/18 (22.2)	0.9 (1.5)	7/34 (20.6)	1.00 ^b^
**13** To know to whom I should direct my questions	1.2 (1.4)	12/52 (23.1)	1.3 (1.3)	4/18 (22.2)	1.2 (1.5)	8/34 (23.5)	1.00 ^b^
**14** To know the probable outcome of my child’s illness	1.8 (1.6)	21/52 (40.4)	1.9 (1.7)	9/18 (50.0)	1.7 (1.6)	12/34 (35.3)	.378 ^b^
**15** To know *how* to give information to my other children (appropriate to his/her age)	1.5 (1.6)	17/46 (37.0)	1.9 (1.7)	10/18 (55.6)	1.3 (1.5)	7/28 (25.0)	.060 ^b^
**16** To know *what* information to give to my other children (appropriate to his/her age)	1.5 (1.5)	15/46 (32.6)	1.8 (1.7)	8/18 (44.4)	1.3 (1.4)	7/28 (25.0)	.208 ^b^
**17** To know how to handle the feelings of my other children	1.7 (1.6)	19/46 (41.3)	1.9 (1.7)	9/18 (50.0)	1.6 (1.6)	10/28 (35.7)	.373 ^b^
**Need for Further Information – additional items**							
**18** To have confidence in the staff caring for my child	1.2 (1.3)	9/53 (17.0)	1.3 (1.1)	2/18 (11.1)	1.1 (1.4)	7/35 (20.0)	.701 ^b^
**19** To know that my child’s pain is well adjusted	1.8 (1.6)	19/51 (37.3)	1.5 (1.6)	5/17 (29.4)	1.9 (1.6)	14/34 (41.2)	.543 ^b^
**20** To be able to speak with my child’s treating physician at any time	1.5 (1.5)	15/53 (28.3)	1.9 (1.5)	7/18 (38.9)	1.3 (1.5)	8/35 (22.9)	.334 ^b^
**21** To have enough time to make decisions	1.2 (1.4)	9/51 (17.6)	1.2 (1.4)	2/18 (11.1)	1.2 (1.5)	7/33 (21.2)	.464 ^b^
**22** To have my child be treated with dignity	1.0 (1.4)	7/50 (14.0)	1.2 (1.6)	3/16 (18.8)	0.9 (1.4)	4/34 (11.8)	.666 ^b^
**23** To have the opportunity to say goodbye to my child	1.2 (1.5)	12/52 (23.1)	1.2 (1.5)	4/17 (23.5)	1.1 (1.6)	8/35 (22.9)	1.00 ^b^
**24** To be with my child when he/she dies	0.9 (1.5)	8/51 (15.7)	0.8 (1.6)	3/17 (17.6)	0.9 (1.5)	5/34 (14.7)	1.00 ^b^
**25** To reach the team 24 hours a day, 7 days a week	0.9 (1.4)	9/52 (17.3)	1.0 (1.6)	3/17 (17.6)	0.8 (1.3)	6/35 (17.1)	1.00 ^b^
**26** To be prepared for the medical aspects of dying by the treating team	1.6 (1.6)	16/53 (30.2)	1.4 (1.6)	5/18 (27.8)	1.6 (1.7)	11/35 (31.4)	1.00 ^b^
**27** To know that my other children were being well cared for as well	1.4 (1.6)	12/47 (25.5)	1.4 (1.5)	5/18 (27.8)	1.3 (1.6)	7/29 (24.1)	1.00 ^b^
**28** To have a contact person for every situation at hand	1.3 (1.5)	11/51 (21.6)	1.4 (1.4)	4/18 (22.2)	1.3 (1.5)	7/33 (21.2)	1.00 ^b^
**29** To have room for conversations with the team about issues other than my child’s disease	1.0 (1.3)	9/52 (17.3)	1.0 (1.4)	4/18 (22.2)	1.0 (1.2)	5/34 (14.7)	.702 ^b^
**30** To be respected for the way I deal with my child’s disease	1.0 (1.4)	9/53 (17.0)	1.0 (1.4)	4/18 (22.2)	1.0 (1.3)	5/35 (14.3)	.469 ^b^
**31** To get support in dealing with different treatment requests within our family	1.3 (1.4)	14/53 (26.4)	1.2 (1.4)	4/18 (22.2)	1.4 (1.5)	10/35 (28.6)	.748 ^b^
**32** To have enough time for myself, e.g. for relaxation	1.3 (1.4)	12/52 (23.1)	1.2 (1.4)	3/18 (16.7)	1.3 (1.4)	9/34 (26.5)	.507 ^b^
**33** To get advice on issues of social-law, such as cost assumption	1.5 (1.6)	16/52 (30.8)	1.2 (1.5)	4/18 (22.2)	1.7 (1.6)	12/34 (35.3)	.529 ^b^

*Abbreviations*: *M* Mean, *SD* Standard deviation

^a^ versus a little/somewhat

^b^ Fisher’s exact test

*p*-values are referring to proportions. * Significance at *p* < 0.05. ** Significance at *p* < 0.01

Need for further information rank: a little (1) to a great deal (4)

Of the 16 additional needs, three needs required very much/a great deal of further information for more than one third of parents: *knowing that the child’s pain is well adjusted* (*M* = 1.8, 37%), *getting advice on issues of social-law* (*M* = 1.6, 31%), and *being prepared for the medical aspects of dying* (*M* = 1.6, 30%). The range of means was 0.9 to 1.8 with standard deviations between 1.3 and 1.6.

Comparing parents of children who died of cancer versus a non-cancer disease, there were no significant differences in the parents` need for further information.

### Use of psychosocial support services

During paediatric palliative care, 61% of parents had accessed at least one psychosocial support service and 84% of parents after the child’s death (Table 5).

**Table 5:** Parent’s utilisation of support services (*N* = 56) and comparison of parents of children with cancer (*N* = 18) and those of children with a non-cancer disease (*N* = 38) during palliative care and after the child’s death

	Overall (*N* = 56)	Parents of children with cancer (*N* = 18)	Parents of children with a non-cancer disease (*N* = 38)	
**During paediatric palliative care**	**n/N (%)**	**n/N (%)**	**n/N (%)**	***p*-value**
Utilisation of ≥1 support service, n(%) yes	34/56 (60.7)	10/18 (55.6)	24/38 (63.2)	.770 ^a^
Support services used (multiple answers possible), n(%) yes				
Self-support group	4/34 (11.8)	1/10 (10.0)	3/24 (12.5)	1.00 ^a^
Cancer counselling service	4/34 (11.8)	4/10 (40.0)	n.a.^b^	n.a.^b^
Psychological counselling	19/34 (55.9)	7/10 (70.0)	12/24 (50.0)	.760 ^a^
Legal counselling	9/34 (26.5)	0/10 (0.0)	9/24 (37.5)	.044* ^a^
Counselling on parenting/family issues	5/34 (14.7)	1/10 (10.0)	4/24 (16.7)	1.00 ^a^
Spiritual counselling	11/34 (32.4)	3/10 (30.0)	8/24 (33.3)	1.00 ^a^
Pre-loss Bereavement care	8/34 (23.5)	1/10 (10.0)	7/24 (29.2)	.411 ^a^
**After the child’s death**	**n/N (%)**	**n/N (%)**	**n/N (%)**	***p*-value**
Utilisation of ≥1 support service, n(%) yes	47/56 (83.9)	14/18 (77.8)	33/38 (86.8)	.448 ^a^
Support services used (multiple answers possible), n(%) yes				
Self-support group	14/47 (29.8)	5/14 (35.7)	9/33 (27.3)	1.00 ^a^
Cancer counselling service	0/47 (0.0)	0/14 (0.0)	n.a.^b^	n.a.^b^
Psychological counselling	31/47 (66.0)	8/14 (57.1)	23/33 (69.7)	.371 ^a^
Legal counselling	0/47 (0.0)	0/14 (0.0)	0/33 (0.0)	n.a.
Counselling on parenting/family issues	3/47 (6.4)	1/14 (7.1)	2/33 (6.1)	1.00 ^a^
Spiritual counselling	13/47 (27.7)	5/14 (35.7)	8/33 (24.2)	.735 ^a^
Bereavement care	19/47 (40.4)	6/14 (42.9)	13/33 (39.4)	1.00 ^b^

^a^Fisher’s Exact Test

^b^not available due to cancer specific support service

^c^Only parents who reported having used support service were included

*Significance at *p* < 0.05, ** Significance at *p* < 0.01

The most frequently used services during palliative care were psychological (56%), spiritual (32%) and legal counselling (27%) and bereavement care (24%).

Comparisons between parents of children who died of cancer versus a non-cancer disease showed significant differences in the utilisation of legal counselling (cancer group: 0%; non-cancer group: 38%; *p* = .044).

After the child’s death, the most frequently accessed services were psychological counselling (66%), bereavement care (40%), self-support groups (30%) and spiritual counselling (28%).

There were no significant differences between the two patient’s groups comparing the parents’ use of psychosocial support services after the patient’s death.

The most frequently reported barriers for accessing support services for all parents were sufficient informal support (38%), no subjective need (23%) and lack of time (20%). Comparisons showed no significant differences between parents of children who died of cancer versus a non-cancer disease (Table 6). Beyond given barriers, two parents reported other reasons for not accessing support services: being afraid of opening up in front of others, and finding it too exhausting to meet parents in a similar situation, as they find their own burden more than enough.

**Table 6:** Barriers for accessing support services (*N* = 56) in parents of children with cancer (*N* = 18) and those of children with a cancer non-disease (*N* = 38)

	Overall (*N* = 56)	Parents of children with cancer (*N* = 18)	Parents of children with a non-cancer disease (*N* = 38)	*p*-value
	n (%)	n (%)	n (%)	
Sufficient informal support	21 (37.5)	7 (38.9)	14 (36.8)	1.00 ^a^
No subjective need	13 (23.2)	6 (33.3)	7 (18.4)	.310 ^a^
Lack of time	11 (19.6)	3 (16.7)	8 (21.1)	1.00 ^a^
Services too far away	8 (14.3)	2 (11.1)	6 (15.8)	1.00 ^a^
Preferring support by treating physicians	6 (10.7)	1 (5.6)	5 (13.2)	.652 ^a^
Lack of knowledge about psychosocial services	4 (7.1)	1 (5.6)	3 (7.9)	1.00 ^a^
No expectation of subjective benefit	4 (7.1)	1 (5.6)	3 (7.9)	1.00 ^a^
Potential burden to family/partnership	3 (5.4)	0 (0.0)	3 (7.9)	.544 ^a^

^a^Fisher’s Exact Test

*Significance at *p* < 0.05. ** Significance at *p* < 0.01

### Relationship between need fulfilment and grief

The average of the ICG-D total score was 26.4 out of 76 points (*SD* = 14.3, range 5–66), and 52% (*n* = 29) of the parents showed noticeable grief symptoms (cut-off ICG-D > 25). In comparison, parents of a child with cancer (*M* = 31.2, *SD* = 16.3) had a higher mean than those of a child with a non-cancer disease (*M* = 24.1, *SD* = 12.9), although this difference was not statistically significant (*p* = .158). Of the parents of children with cancer, 67% (*n* = 12) showed noticeable grief symptoms and 45% (*n* = 17) of the parents of children with non-cancer diseases.

We investigated correlations between the fulfilment of single needs and the mean total score of complicated grief (see Table 7). Overall, one need was significantly negatively correlated to the grief symptoms with a small to medium effect size [36]: *having the opportunity to say goodbye to the child* (*r* = −.278, *p* = .042).

**Table 7:** Correlations between need fulfilment (FIN-PED II) and level of grief (ICG-D)

FIN-PED II - Need Fulfilment	Level of grief symptoms (ICG-D)		
	Overall (*N* = 56)	Parents of children with cancer (*N* = 18)	Parents of children with a non-cancer disease (*N* = 38)
**1** To feel there was hope	*r* = −.189	*r* = .050	*r* = −.296
	*p* = .220	*p* = .866	*p* = .113
**2** To know when to expect side effects to occur	*r* = .115	*r* = .039	*r* = .117
	*p* = .416	*p* = .881	*p* = .504
**3** To know what side effects the treatment can cause	*r* = .068	*r* = −.095	*r* = .194
	*p* = .627	*p* = .717	*p* = .256
**4** To have thorough information about how to care for my child at home	*r* = .067	*r* = .139	*r* = .010
	*p* = .627	*p* = .581	*p* = .953
**5** To know that health care professionals offer me the opportunity to participate equally in my child’s care	*r* = .049	*r* = .497*	*r* = −.170
	*p* = .725	*p* = .042	*p* = .322
**6** To have trust in the health care system	*r* = .122	*r* = .213	*r* = .089
	*p* = .393	*p* = .412	*p* = .617
**7** To be informed of changes to my child’s condition	*r* = −.108	*r* = −.238	*r* = −.018
	*p* = .432	*p* = .343	*p* = .915
**8** To know what treatment my child was receiving	*r* = −.088	*r* = .084	*r* = −.157
	*p* = .521	*p* = .741	*p* = .354
**9** To feel that the health care professionals were sincere in caring about my child	*r* = −.207	*r* = −.151	*r* = −.159
	*p* = .125	*p* = .550	*p* = .340
**10** To have explanations given in terms that were understandable to me	*r* = .028	*r* = .180	*r* = .066
	*p* = .843	*p* = .475	*p* = .704
**11** To be told when and when and why changes were being made in my child’s treatment plans	*r* = .134	*r* = .189	*r* = .216
	*p* = .350	*p* = .484	*p* = .213
**12** To know I could ask questions any time	*r* = −.158	*r* = −.077	*r* = −.108
	*p* = .249	*p* = .761	*p* = .525
**13** To know to whom I should direct my questions	*r* = −.147	*r* = −.169	*r* = −.073
	*p* = .284	*p* = .503	*p* = .666
**14** To know the probable outcome of my child’s illness	*r* = .185	*r* = .035	*r* = .295
	*p* = .181	*p* = .890	*p* = .081
**15** To know how to give information to my other children (appropriate to his/her age)	*r* = −.162	*r* = .238	*r* = −.493*
	*p* = .325	*p* = .393	*p* = .014
**16** To know what information to give to my other children (appropriate to his/her age)	*r* = −.195	*r* = .168	*r* = −.523*
	*p* = .240	*p* = .549	*p* = .010
**17** To know how to handle the feelings of my other children	*r* = −.171	*r* = .197	*r* = −.454*
	*p* = .291	*p* = .481	*p* = .023
**Need Fulfilment – additional items**			
**18** To have confidence in the staff caring for my child	*r* = −.180	*r* = −.233	*r* = −.139
	*p* = .194	*p* = .352	*p* = .419
**19** To know that my child’s pain is well adjusted	*r* = .095	*r* = .038	*r* = .071
	*p* = .494	*p* = .881	*p* = .680
**20** To be able to speak with my child’s caring physician at any time	*r* = −.033	*r* = .283	*r* = −.116
	*p* = .815	*p* = .255	*p* = .499
**21 **To have enough time to make decisions	*r* = .026	*r* = .052	*r* = .068
	*p* = .854	*p* = .849	*p* = .699
**22** To have my child be treated with dignity	*r* = −.185	*r* = −.183	*r* = −.151
	*p* = .177	*p* = .466	*p* = .371
**23** To have the opportunity to say goodbye to my child	*r* = −.278*	*r* = −.028	*r* = −.375*
	*p* = .042	*p* = .914	*p* = .022
**24** To be with my child when he/she dies	*r* = −.177	*r* = −.408	*r* = −.188
	*p* = .201	*p* = .093	*p* = .273
**25** To reach the team 24 hours a day, 7 days a week	*r* = −.120	*r* = −.073	*r* = −.120
	*p* = .384	*p* = .773	*p* = .480
**26** To be prepared for the medical aspects of dying by the treatment team	*r* = −.182	*r* = −.127	*r* = −.237
	*p* = .187	*p* = .615	*p* = .164
**27** To know that my other children were being well cared for as well	*r* = −.092	*r* = .014	*r* = −.174
	*p* = .574	*p* = .961	*p* = .405
**28** To have a contact person for every situation at hand	*r* = −.100	*r* = .137	*r* = −.205
	*p* = .484	*p* = .589	*p* = .253
**29** To have room for conversations with the team about issues other than my child’s disease	*r* = .130	*r* = .463	*r* = −.069
	*p* = .406	*p* = .082	*p* = .727
**30** To be respected for the way I deal with my child’s disease	*r* = −.151	*r* = −.308	*r* = −.058
	*p* = .276	*p* = .229	*p* = .734
**31** To get support in dealing with different treatment requests within our family	*r* = .043	*r* = .310	*r* = −.084
	*p* = .786	*p* = .280	*p* = .665
**32** To have enough time for myself, e.g. for relaxation	*r* = −.162	*r* = −.199	*r* = −.180
	*p* = .326	*p* = .496	*p* = .390
**33 **To get advice on issues of social-law, such as cost assumption	*r* = −.084	*r* = .116	*r* = −.238
	*p* = .564	*p* = .669	*p* = .182

*Abbreviations*: *r* Pearson’s correlation coefficient

* Significance at *p* < 0.05. ** Significance at *p* < 0.01

In parents of a child with cancer, one need was significantly positively correlated to the grief symptoms with a large effect size: *knowing that the health care professionals offer the opportunity to participate equally in the child’s care”* (*r* = .497, *p* = .042). The more the need was fulfilled, the higher were the grief symptoms.

In parents of a child with a non-cancer disease, four needs were significantly negatively correlated with a medium to large effect size: *knowing how (r* = −.493, *p* = .014*) and what (r* = −.523, *p* = .010*) information to give to siblings*, *knowing how to handle the siblings’ feelings* (*r* = −.454, *p* = .023) and *having the opportunity to say goodbye to the child* (*r* = −.375, *p* = .022). The less the needs were fulfilled, the higher were the grief symptoms.

## Discussion

In this exploratory study, we report on supportive care needs and the utilisation of psycho-social support services among parents of children who had died and had received specialist paediatric palliative care, as well as on the relationship between need fulfilment and grief. We further explored possible differences between parents whose child died of cancer versus those who died of a non-cancer related disease.

A relevant finding of our study is that many needs reported as most important were considered as being met, regardless of the underlying disease of the deceased child. In contrast, a study by Kassam et al. [5] reported that the needs highly valued by parents, such as communication aspects of care, were reported less met. In our study, highest ranked needs concerned: being able to ask questions at any time, being informed of changes in the child’s condition, and knowing healthcare professionals are sincere about caring. Thus, the most important needs focused on clear, honest communication and the family’s reassurance of compassionate caring. Sensitive caring as well as honest and prognostic conversations have been identified to correlate with parental perception of the quality of paediatric palliative care [37], in addition to better coping and less grief [38]. As Ekberg et al. [39] pointed out, effective communication of healthcare professionals is a core component of competence in palliative care.

Our study showed the high relevance for support offered to siblings. Previous literature identified sibling care as a relevant area of need among parents with children who have life-limiting diseases [5, 6], because parents are challenged to balance the care and commitment for their ill child with the needs of their other children [40]. However, such needs often remain unmet, as observed both in our study (about 40–55% of parents) and in previous research [4]. As it has already been concluded, approaches to psychosocial care support in paediatric palliative care should include sibling care as a relevant task to relieve the burden on families affected [37]. Although not statistically significant, unmet needs related to sibling care were more frequent in parents of children with cancer. While in most non-cancer related families the illness is part of everyday life since birth, children with cancer were healthy for some part of their life. Further, studies indicate that referral to specialist paediatric palliative care occurs late in the illness trajectory among children with cancer, sometimes only days before death [41, 42]. Therefore, the need for supporting siblings could possibly be more urgent and overwhelming in these parents.

Another need of parents concerned the feeling hope; yet, two thirds of parents reported this need as unmet. “Hope” was not characterized in the FIN-PED II; thus, it remains unclear, which kind of hope parents were referring to when rating this need. Previous studies propose that harboring hope for cure may be a coping strategy of parents facing a palliative diagnosis of their child [43, 44]. Kassam et al. [5] showed that when compared with physicians, parents of children with cancer gave a higher importance rating to cancer-directed therapy. Mack et al. [45] found that, retrospectively, about 55% of the parents would have changed their goal of palliative cancer targeted therapy. Maintaining hope, as a coping strategy, should be in the awareness of paediatric palliative care teams, but also the fact that parents might regret decisions later. However, evidence shows that parental hope is “life sustaining, essential, and a constant even in the face of lingering despair” [46]. As it is a multi-facetted phenomenon, various forms of hope exist and hopes may change during the parent’s difficult journey [43, 47, 48]. Therefore, it is of importance that health care professionals extrapolate what parents are hoping for, what maintaining or giving up hope means for these parents, and how a supportive environment for hope can be created.

Beyond parental needs, we investigated parent’s utilisation of professional psychosocial support. Notably, about 60% of the parents had accessed at least one psychosocial support service during specialist paediatric palliative care, and 84% after the child’s death. While parents of children with cancer did not use support in legal matters, a quarter of parents of children with a non-cancer disease did. Monterosso et al. [17] and Zimmermann et al. [49] pointed out that for families caring for a child with cancer most hospitals provide counselling on social-law issues as standard care. The most prominent barriers for accessing services were: sufficient informal support, no subjective need, and lack of time. To reach families, a better understanding of the need and benefit of support services could help parents to assess whether additional professional support would be useful. Health care professionals should facilitate pathways to psychosocial care by sustained provision of information.

A further aim of our study was to investigate a possible relationship between the fulfilment of needs and symptoms of grief. A higher level of grief symptoms significantly correlated with a lower fulfilment of having the option to say goodbye to the child with a medium correlational effect, but no further significant correlation could be found. Meaningful communication, including saying goodbye to the child, has been shown to be of importance in terms of post-loss outcomes: Having said goodbye was significantly associated with lower levels of the parent’s grief [50, 51]. Thus, supporting parent’s experience of saying goodbye to the child may reduce long-term distress during bereavement. Saying goodbye however, may be achieved in different ways (e.g., in words, symbolically) [50]. Health care professionals, being aware of this important need, may support parents in finding their own individual way if needed.

We observed a significant positive correlation between higher levels of grief and the fulfilment of knowing about the opportunity to participating equally in the child’s care in bereaved parents of children with cancer, but not in those of children with a non-cancer disease. One possible explanation could be that the latter were more used to participate equally in the child’s care due to the previous life-long care for their chronically ill child. Faced with an acute disease, parents caring for a child with cancer may feel less competent. In parents of children who died of a non-cancer disease, three of the four needs that were negatively correlated with higher levels of grief concerned sibling care. This finding is supported by a study that found a relationship between being able to talk about feelings in the family and lower levels of prolonged grief symptoms for mothers [51]. Feeling able to inform and comfort siblings to one’s satisfaction might promote better coping with the experienced loss.

### Limitations

Limitations of this study concern the generalisability and transferability of the results.

The sample size was small and the response rate low (36%). However, the response rate is compatible to other empirical research in paediatric palliative care [2], and it is well known that recruitment for studies in bereaved parents presents unique challenges [52]. Findings might be biased in that more strongly affected parents might have been motivated to participate, which might lead to an overestimation of negative effects. On the other hand, extremely heavily affected parents might not have responded at all, which might lead to an underestimation. Most respondents (75%) were mothers, which corresponds with other studies in this field, since the mothers are usually reported to be the dominant response group [4, 17, 24, 53, 54]. Also, this could reflect the common situation in Germany, where mothers, who have young children are less likely to be employed [55]. Lastly, this study was conducted with a homogenous population in the context of specialist paediatric palliative care in the cultural setting of Germany and may not be generalised to other populations.

Further, there might be a recall-bias concerning the retrospective evaluation of needs, as the time since the child’s death varies between 6 months to 8 years. Retrospective perspectives might change and vary from the acute experiences during paediatric palliative care. Conventional tests of significance were used despite the small sample size, which could compromise the conclusions drawn from the study. The strengths of this study include the use of standardised instruments to assess parental support needs, which are rarely applied in paediatric palliative care research, as indicated by a recent review [4]. This is the first study in Germany using the FIN PED II. Nevertheless, Cronbach’s alpha for our extended version of the questionnaire has to be critically discussed, as a high value of alpha (>.90) may suggest redundancies and indicates to reduce the number of items [56]. Although validation of the extended version was not the aim of this study, as the added items solely were important additions from practical experience, it has to be kept in mind that these items do not generally improve the questionnaire.

## Conclusions

Setting standards for paediatric palliative care that enable to adequately address the needs for parents of children suffering from cancer or a non-cancer disease should be a basic goal for every paediatric palliative care team. This study offers guidance for enhancing the fulfilment of parental needs in paediatric palliative care by identifying unmet needs as well as needs for which further information might be required. The study could show that there should be a focus on the feeling and sharing of hope. Furthermore, supporting the communication with siblings and strengthening the parents by providing communication tools is not only important during the dying process itself, but also to prevent complicated grief. To enhance early identification of parental needs and offer of adequate support, paediatric palliative care should implement routine assessment of needs. In future studies, focus should be set on gathering information about needs at more than one time for example at diagnosis, end of treatment, recurrence or progress of the underlying disease and death.

## Data Availability

The datasets generated and/or analysed during the current study are not publicly available due to reasons of privacy but are available from the corresponding author (AB) on reasonable request.
